# Serum osteoprotegerin is an independent marker of central arterial stiffness as assessed using carotid–femoral pulse wave velocity in hemodialysis patients: a cross sectional study

**DOI:** 10.1186/s12882-019-1374-2

**Published:** 2019-05-23

**Authors:** Jia-Sian Hou, Yu-Li Lin, Chih-Hsien Wang, Yu-Hsien Lai, Chiu-Huang Kuo, Yi-Maun Subeq, Bang-Gee Hsu

**Affiliations:** 1 Division of Nephrology, Buddhist Tzu Chi General Hospital, Hualien, Taiwan; 2 Institute of Medical Sciences, Tzu Chi University, Hualien, Taiwan; 3 Department of Nursing, National Taichung University of Science and Technology, Hualien, Taiwan; 4 School of Medicine, Tzu Chi University, Hualien, Taiwan

**Keywords:** Osteoprotegerin, Arterial stiffness, Carotid-femoral pulse wave velocity, Hemodialysis

## Abstract

**Background:**

Cardiovascular morbidity and mortality are highly prevalent in patients with end-stage renal disease, and osteoprotegerin (OPG) may be an important link between bone loss and vascular calcification. This study was conducted to evaluate the relationship between central arterial stiffness and serum OPG levels in hemodialysis (HD) patients.

**Methods:**

Blood samples were collected from 120 HD patients, and the carotid–femoral pulse wave velocity (cfPWV) value was measured using a validated tonometry system. The cfPWV value of > 10 m/s was used to define the high artery stiffness group. Serum OPG levels were analyzed categorically into tertiles.

**Results:**

Of the 120 HD patients, 53 (44.2%) were defined as the high arterial stiffness group, who had higher values of systolic blood pressure (*p* = 0.038), serum calcium (*p* = 0.007), and OPG (*p* <  0.001) levels and a higher prevalence of diabetes mellitus (DM, *p* = 0.001). Increasing tertiles of serum OPG levels were significantly associated with greater height (*p* = 0.011), male gender (*p* = 0.008), higher cfPWV values (*p* = 0.020), and lower intact parathyroid hormone (iPTH, *p* = 0.049) levels. Multivariable linear regression analysis showed that cfPWV value was independently associated with DM (β = 1.83, *p* = 0.008) and increasing tertiles of serum OPG levels (β = 0.89 and 1.63 for tertile 2 and tertile 3, respectively, *p* for trend = 0.035) in HD patients. Multivariable logistic regression analysis revealed that, in addition to age, DM, low iPTH levels, and high serum calcium levels, increasing tertiles of serum OPG levels (OR = 5.34 for tertile 2; OR = 7.06 for tertile 3; *p* for trend = 0.002) were an independent predictor of high arterial stiffness in HD patients. Serum calcium levels positively correlated with cfPWV value only in the highest OPG tertile group (*r* = 0.408, *p* = 0.009).

**Conclusion:**

A positive association was detected between serum OPG levels and central arterial stiffness in HD patients, and patients with high serum OPG levels may have greater influence of calcium load on central arterial stiffening.

## Background

Cardiovascular (CV) disease is highly prevalent in patients with end-stage renal disease (ESRD), accounting for approximately 20–60% of all-cause death [1–3]. In addition to traditional risk factors for atherosclerosis, patients with chronic kidney disease (CKD) and ESRD are susceptible to vascular calcification, which is characterized by medial and intimal layer calcification [4, 5]. Both the abovementioned conditions subsequently contribute to increased arterial stiffness and risk of premature CV disease death. Thus, exploring the potential mechanisms of vascular calcification in patients with ESRD is of paramount importance. Aging, inflammation, oxidative stress, diabetes mellitus (DM), and calcium and phosphorus loading are well-established factors associated with vascular calcification [5, 6]. Moreover, cumulative evidence has demonstrated that abnormal bone mineralization is also involved in the pathogenesis of vascular calcification.

Osteoprotegerin (OPG), a soluble glycoprotein that binds to receptor activator of nuclear factor kappa-B ligand (RANKL) and TNF-related apoptosis-inducing ligand (TRAIL), is not only well-known to play a critical role in bone homeostasis, but it has also been implicated in the complex process of vascular remodeling. The stimulation of OPG/RANK/RANKL signaling, through inflammation and reactive oxygen species, has been reported to be involved in the pathogenesis of vascular calcification [7]. This connection is of particular interest in patients with ESRD due to the accelerated vascular calcification and the markedly elevated plasma OPG levels in this special population [8]. However, this complex pathogenesis has not yet been completely understood.

Carotid–femoral pulse wave velocity (cfPWV) is a noninvasive gold standard method for measuring large artery stiffness [9, 10], which is known to be associated with CV mortality in patients with ESRD [11]. In this study, we determined whether serum OPG levels are associated with central arterial stiffness in chronic hemodialysis (HD) patients, independent of the abovementioned well-established vascular risk factors.

## Methods

### Patients

The cross-sectional study was conducted at a medical center in Hualien between March and July in 2015 and was approved by the Institutional Review Board of Tzu-Chi Hospital. Patients aged more than 20 years and who had undergone HD for at least 3 months with standard 4-h dialysis three times per week were included. The same type of high-flux polysulfone disposable artificial kidney (FX class dialyzer, Fresenius Medical Care, Bad Homburg, Germany) was used by all patients. Patients who had any infection, acute myocardial infarction, stroke, peripheral arterial occlusive diseases, pulmonary edema, arrhythmias and atrial fibrillation at the time of enrollment or who refused to participate were excluded from the study. DM was defined based on a history of antidiabetic drug use, and hypertension was defined based on a history of or receiving antihypertensive agents. Coronary artery disease (CAD) was diagnosed through coronary angiography. The drug use history was collected by a review of medical records, which included the use of angiotensin-converting enzyme inhibitors (ACEIs), angiotensin receptor blockers (ARBs), calcium channel blockers (CCBs), β-blockers, and statins.

### Anthropometric analysis and blood pressure measurement

Body weights were measured in light clothing after HD, and height was measured to the nearest half centimeter without shoes. Body mass index (BMI) was calculated as body weight (kilograms) divided by height squared (meters). Blood pressure was measured before the HD session using standard mercury sphygmomanometers.

### Measurement of arterial stiffness

PWV was evaluated from the time taken for the arterial pulse waveform to propagate from the carotid to the femoral artery, using applanation tonometry (SphygmoCor system, AtCor Medical, Australia). A transcutaneous recording of the pressure pulse waveform in the underlying artery was performed in the supine position after a minimum 10-min rest before the HD session. The cfPWV was calculated using the distance and mean time difference between the two superficial artery sites. A detailed explanation of this device has been provided elsewhere [12]. Patients with cfPWV > 10 m/s were defined as the high arterial stiffness group, according to the European Society of Hypertension and the European Society of Cardiology (ESH-ESC) 2013 Guidelines [10].

### Biochemical determinations

Blood samples were collected from the patients for analysis before the HD session. Fasting blood samples (approximately 5 mL) were immediately centrifuged at 3000 g for 10 min. The obtained serum samples were stored at 4 °C and used for biochemical analyses within 1 h of collection. Serum levels of blood urea nitrogen (BUN), creatinine, glucose, total cholesterol, calcium, phosphorus, and C-reactive protein were measured using an autoanalyzer (Siemens Advia 1800, Siemens Healthcare GmbH, Henkestr, Germany). Corrected serum calcium levels were calculated as follows: corrected calcium (mg/dL) = serum calcium (mg/dL) + 0.8 [4 – serum albumin (mg/dL)], and the upper normal limit was chosen as the reference value according to the K/DOQI recommendations [13]. The levels of intact parathyroid hormone (i-PTH) were measured by an autoanalyzer (Diagnostic Systems Laboratories, Webster, Texas, USA), and levels less than two times the upper limit and more than nine times the upper normal limit were defined as low and high parathyroid hormone levels, which are 130 and 595 pg/mL, respectively [14]. Serum OPG levels were measured using a commercially available enzyme-linked immunosorbent assays (ELISA) (eBioscience Inc., San Diego, CA, USA). The fractional clearance index for urea (Kt/V) was measured before and immediately after dialysis using a formal, single-compartment dialysis urea kinetic model.

### Statistical analysis

The normality of the distributions of continuous variables was assessed using the Kolmogorov–Smirnov test. Variables with normal distribution were expressed as mean ± standard deviation and analyzed by the Student’s independent t-test, whereas those not normally distributed were expressed as medians and interquartile ranges and analyzed by the Mann–Whitney U test. Categorical variables were expressed as absolute (n) and relative frequency (%) and analyzed by the χ^2^ test or Fisher’s exact test. Serum OPG levels were analyzed categorically into tertiles, and characteristics of the study population in different OPG groups were evaluated for linear trend using one-way analysis of variance (ANOVA) or the Cochran–Armitage test for trend. The values of cfPWV, HD duration, glucose levels, iPTH levels, and serum OPG levels showed skewed distribution and were logarithmically transformed to achieve normality. Univariable correlations between cfPWV and potential explanatory variables were assessed by Pearson’s correlation coefficient. Multivariable linear and logistic regression analyses were conducted using cfPWV values and the presence of high arterial stiffness (defined as cfPWV > 10 m/s) as dependent variables and potential predictors (age, sex, DM, systolic blood pressure, corrected serum calcium, phosphorus, iPTH, and OPG levels) as independent variables. Data were analyzed using SPSS for Windows (version 19.0; SPSS Inc., Chicago, IL, USA). A *p* value < 0.05 was considered as statistically significant.

## Results

### Population characteristics

A total of 120 chronic HD patients were enrolled in our study. The mean age of the patients was 63.3 ± 13.7 years, and 53 (44.2%) of them had high arterial stiffness. The clinical and laboratory characteristics of the 120 chronic HD patients with low or high arterial stiffness are presented in Table 1. Compared with the low arterial stiffness group, patients in the high arterial stiffness group had higher systolic blood pressure (*p* = 0.038), cfPWV (*p* <  0.001), corrected serum calcium (*p* = 0.007), and OPG levels (*p* <  0.001). In addition, the proportion of DM was higher in the high arterial stiffness group (*p* = 0.001).

**Table 1:** Clinical variables of the 120 patients with low or high arterial stiffness

Characteristics	All Patients (*n* = 120)	Low Arterial Stiffness Group (*n* = 67)	High Arterial Stiffness Group (*n* = 53)	*p* value
Demographics				
Age (years)	63.3 ± 13.7	61.7 ± 14.1	65.4 ± 13.2	0.144
Female gender	60 (50.0)	35 (52.2)	25 (47.2)	0.851
HD duration (years)	4.7 (2.0–9.8)	5.8 (1.8–11.1)	4.6 (2.3–7.1)	0.434
Examination				
Height (cm)	159.6 ± 8.5	158.9 ± 8.9	160.6 ± 8.0	0.290
Body weight (kg)	61.5 ± 14.6	63.3 ± 16.3	65.5 ± 13.8	0.444
BMI (kg/m^2^)	25.1 ± 5.0	24.9 ± 5.4	25.3 ± 4.5	0.681
SBP (mmHg)	142.2 ± 25.2	137.9 ± 26.3	147.5 ± 22.8	0.038*
DBP (mmHg)	76.8 ± 16.6	76.2 ± 15.5	77.5 ± 18.0	0.669
cfPWV (m/s)	9.30 (7.63–12.37)	7.80 (7.10–8.80)	12.6 (11.40–15.25)	< 0.001*
Laboratory data				
Total cholesterol (mg/dL)	144.2 ± 32.3	145.0 ± 35.8	143.2 ± 27.6	0.582
Glucose (mg/dL)	131.5 (110.3–172.8)	129.0 (110.0–163.0)	134.0 (110.5–185.0)	0.235
BUN (mg/dL)	61.9 ± 14.5	61.9 ± 13.1	61.9 ± 16.2	0.978
Creatinine (mg/dL)	9.2 ± 2.0	9.3 ± 2.0	9.0 ± 2.0	0.397
Corrected calcium (mg/dL)	8.8 ± 0.6	8.6 ± 0.7	9.0 ± 0.8	0.007*
Phosphorus (mg/dL)	4.8 ± 1.2	4.8 ± 1.3	4.9 ± 1.2	0.573
C-reactive protein (mg/dL)	0.31 (0.08–0.89)	0.30 (0.08–1.01)	0.34 (0.09–0.74)	0.830
Intact PTH (pg/mL)	190.0 (58.5–355.3)	239.3 (114.4–372.5)	141.1 (44.4–339.9)	0.066
OPG (pg/L)	286.2 (209.0–343.6)	247.9 (187.8–332.2)	316.3 (246.2–413.3)	< 0.001*
Kt/V (Gotch)	1.34 ± 0.17	1.35 ± 0.18	1.34 ± 0.16	0.717
Comorbid conditions				
Diabetes mellitus, *s* (%)	56 (46.7)	22 (32.8)	33 (64.2)	0.001*
Hypertension, *n* (%)	60 (50.0)	29 (43.3)	31 (58.5)	0.098
CAD, *n* (%)	78 (65.0)	39 (58.2)	39 (73.6)	0.079
Medications				
ACEIs or ARBs, *n* (%)	41 (34.2)	21 (31.3)	20 (37.7)	0.463
β-Blockers, *n* (%)	33 (20.6)	19 (28.4)	14 (26.4)	0.813
CCBs, *n* (%)	44 (27.5)	27 (40.3)	17 (32.1)	0.353
Statins, *n* (%)	17 (14.2)	7 (10.4)	10 (18.9)	0.189

Values for continuous variables are shown as mean ± standard deviation and analyzed by Student’s *t*-test; variables not normally distributed are shown as median and interquartile range and analyzed by the Mann–Whitney U test; values for categorical variables are presented as number (%) and analyzed by the chi-square test or Fisher’s exact test

*HD* hemodialysis, *BMI* body mass index, *SBP* systolic blood pressure, *DBP* diastolic blood pressure, *cfPWV* carotid–femoral pulse wave velocity, *BUN* blood urea nitrogen, *PTH* parathyroid hormone, *OPG* osteoprotegerin, *Kt/V* fractional clearance index for urea, *CAD* coronary artery disease, *ACEIs* angiotensin-converting enzyme inhibitors, *ARBs* angiotensin receptor blockers, *CCBs* calcium channel blockers

*p < 0.05 is considered as statistically significant

The clinical characteristics of the study population were stratified by tertiles of serum OPG levels (Table 2). Increasing tertiles of serum OPG levels showed a significant association with greater height (*p* = 0.011), male gender (*p* = 0.008), higher cfPWV (*p* = 0.020), and lower iPTH levels (*p* = 0.049).

**Table 2:** Clinical characteristics of the study population stratified by tertiles of serum OPG levels

Characteristics	Tertile 1 (72.97–237.13 pg/L) (*n* = 40)	Tertile 2 (237.36–323.54 pg/L) (*n* = 40)	Tertile 3 (329.61–835.78 pg/L) (*n* = 40)	*p* for Trend
Demographics				
Age (years)	63.6 ± 12.9	63.3 ± 15.0	63.0 ± 15.6	0.834
Female gender	25 (62.5)	22 (55.0)	13 (32.5)	0.008*
HD duration (years)	4.5 (1.5–11.7)	5.1 (2.3–9.1)	4.7 (2.3–10.1)	1.000
Examination				
Height (cm)	157.8 ± 6.6	158.5 ± 9.4	162.6 ± 8.8	0.011*
Body weight (kg)	63.9 ± 15.1	58.8 ± 13.2	70.1 ± 15.4	0.060
BMI (kg/m^2^)	25.6 ± 5.7	23.3 ± 4.2	26.3 ± 4.6	0.521
SBP (mmHg)	143.1 ± 27.2	145.0 ± 23.8	138.4 ± 24.7	0.414
DBP (mmHg)	75.9 ± 15.4	78.6 ± 19.0	75.8 ± 15.4	0.989
cfPWV (m/s)	8.45 (7.53–10.05)	10.20 (7.53–12.35)	10.45 (7.80–13.23)	0.020*
Laboratory data				
Total cholesterol (mg/dL)	150.1 ± 35.2	142.0 ± 32.4	140.6 ± 29.1	0.193
Glucose (mg/dL)	130.5 (113.3–168.8)	127.0 (107.0–150.3)	136.5 (106.0–212.0)	0.290
BUN (mg/dL)	61.6 ± 13.6	62.5 ± 14.8	61.5 ± 15.4	0.976
Creatinine (mg/dL)	9.0 ± 1.7	9.2 ± 2.3	9.4 ± 1.9	0.378
Corrected calcium (mg/dL)	8.9 ± 0.8	8.8 ± 0.8	8.8 ± 0.8	0.424
Phosphorus (mg/dL)	5.0 ± 1.4	4.8 ± 1.1	4.7 ± 1.2	0.279
C-reactive protein (mg/dL)	0.49 (0.10–1.32)	0.22 (0.08–0.46)	0.34 (0.08–1.12)	0.385
Intact PTH (pg/mL)	250.9 (137.0–534.0)	146.5 (56.9–338.2)	160.4 (58.1–342.2)	0.049*
Kt/V (Gotch)	1.35 ± 0.16	1.37 ± 0.15	1.31 ± 0.20	0.224
Comorbid conditions				
Diabetes mellitus, *n* (%)	20 (50.0)	19 (47.5)	17 (42.5)	0.503
Hypertension, *n* (%)	19 (47.5)	20 (50.0)	21 (52.5)	0.656
CAD, n (%)	30 (75.0)	22 (55.0)	26 (65.0)	0.169
Medications				
ACEIs or ARBs, *n* (%)	13 (32.5)	12 (30)	17 (42.5)	0.396
β-Blockers, *n* (%)	13 (32.5)	12 (30)	8 (20)	0.212
CCBs, *n* (%)	17 (42.5)	15 (37.5)	12 (30.0)	0.248
Statins, n (%)	4 (10)	7 (17.5)	6 (15)	0.619

Values for continuous variables are shown as mean ± standard deviation; variables not normally distributed are shown as median and interquartile range; values for categorical variables are presented as number (%). characteristics of the study population in different OPG groups were evaluated for linear trend using one-way analysis of variance (ANOVA) or the Cochran–Armitage test for trend

*OPG* osteoprotegerin, *HD* hemodialysis, *BMI* body mass index, *SBP* systolic blood pressure, *DBP* diastolic blood pressure, *cfPWV* carotid–femoral pulse wave velocity, *BUN* blood urea nitrogen, *PTH* parathyroid hormone, *Kt/V* fractional clearance index for urea, *CAD* coronary artery disease, *ACEIs* angiotensin-converting enzyme inhibitors, *ARBs* angiotensin receptor blockers; CCBs, calcium channel blockers

*p < 0.05 is considered as statistically significant

### Factors correlated with cfPWV

The continuous variables were found to correlate with cfPWV values among the 120 HD patients, as shown in Table 3. The cfPWV values showed a positive correlation with systolic blood pressure (*r* = 0.196, *p* = 0.032), serum glucose (*r* = 0.182, *p* = 0.047), and OPG levels (*r* = 0.281, *p* = 0.002).

**Table 3:** Correlation between cfPWV values and clinical variables among 120 hemodialysis patients

Variables	Log-cfPWV (m/s)	
	*r*	*p* value
Age (years)	0.099	0.280
Log-HD duration (months)	− 0.070	0.445
Height (cm)	0.121	0.187
Body weight (kg)	0.101	0.272
BMI (kg/m^2^)	0.061	0.506
SBP (mmHg)	0.196	0.032*
DBP (mmHg)	0.038	0.678
Total cholesterol (mg/dL)	−0.056	0.542
Log-Glucose (mg/dL)	0.182	0.047*
BUN (mg/dL)	0.040	0.662
Creatinine (mg/dL)	−0.030	0.746
Corrected calcium (mg/dL)	0.128	0.165
Phosphorus (mg/dL)	0.072	0.434
Log-CRP (mg/dL)	0.061	0.506
Log-iPTH (pg/mL)	−0.146	0.112
Log-OPG (pg/L)	0.281	0.002*
Kt/V (Gotch)	−0.030	0.747

The data of cfPWV, HD duration, glucose, iPTH and OPG showed skewed distribution and were log-transformed before analysis

Data were analyzed using Pearson’s correlation analysis

*cfPWV* carotid–femoral pulse wave velocity, *HD* hemodialysis, *BMI* body mass index, *SBP* systolic blood pressure, *DBP* diastolic blood pressure, *BUN* blood urea nitrogen, *iPTH* intact parathyroid hormone, *CRP* C-reactive protein, *OPG* osteoprotegerin, *Kt/V* fractional clearance index for urea

*p < 0.05 is considered as statistically significant

The factors associated with cfPWV values were analyzed among the 120 HD patients. After multivariable adjustment, increasing tertiles of serum OPG levels (β = 0.89 and 1.63 for tertile 2 and tertile 3, respectively, *p* for trend = 0.035) and the presence of DM (β = 1.83, *p* = 0.008) were the two independent factors that were associated with cfPWV values. Notably, low iPTH levels were also marginally associated with cfPWV values (β = 1.29, *p* = 0.055), compared to iPTH levels in the normal range (Table 4).

**Table 4:** Multivariable linear regression analysis of the factors that correlated with cfPWV values among 120 hemodialysis patients

Variables	β (95% confidence interval)	*p* value
OPG group		
Tertile 1	Reference	0.035*^¶^
Tertile 2	0.89 (−0.61–2.39)	
Tertile 3	1.63 (0.11–3.15)	
Diabetes		
No	Reference	
Yes	1.83 (0.48–3.18)	0.008*
Intact PTH levels (pg/mL)		
Normal (130–595 pg/mL)	Reference	
Low (< 130 pg/mL)	1.29 (−0.03–2.61)	0.055
High (> 595 pg/mL)	1.22 (− 0.96–3.40)	0.270

Adopted factors: age, sex, diabetes, systolic blood pressure, corrected serum calcium, phosphorus, intact parathyroid hormone, glucose, C-reactive protein, and osteoprotegerin levels

Adjusted R^2^ = 0.160

*cfPWV* carotid–femoral pulse wave velocity, *OPG* osteoprotegerin, *PTH* parathyroid hormone

^¶^*p* for trend

*p < 0.05 is considered as statistically significant

### Factor associated with arterial stiffness with multivariable logistic regression analysis

Table 5 delineates the results of the multivariable logistic regression analysis regarding the factors associated with high arterial stiffness. Age (odds ratio, [OR] = 1.04, 95% confidence interval [95% CI] = 1.01–1.08, *p* = 0.026), increasing tertiles of serum OPG levels (OR = 5.34 for tertile 2; OR = 7.06 for tertile 3; *p* for trend = 0.002), DM (OR = 3.65, 95% CI = 1.34–9.98, *p* = 0.012), low iPTH levels (OR = 2.83, 95% CI = 1.06–7.53, *p* = 0.038), and high corrected calcium levels (OR = 3.73, 95% CI = 1.16–11.98, *p* = 0.027) were independently associated with high arterial stiffness.

**Table 5:** Multivariable logistic regression analysis of the factors associated with high arterial stiffness among 120 hemodialysis patients

Variables	Odds ratio (95% confidence interval)	*p* value
Age (years)	1.04 (1.01–1.08)	0.026*
OPG group		
Tertile 1	Reference	0.002*^¶^
Tertile 2	5.34 (1.63–17.42)	
Tertile 3	7.06 (2.03–24.49)	
Diabetes		
No	Reference	0.012*
Yes	3.65 (1.34–9.98)	
Intact PTH levels (pg/mL)		
Normal (130–595 pg/mL)	Reference	
Low (< 130 pg/mL)	2.83 (1.06–7.53)	0.038*
High (> 595 pg/mL)	2.89 (0.59–14.15)	0.190
Corrected calcium (mg/dL)		
< 9.5 mg/dL	Reference	0.027*
≥ 9.5 mg/dL	3.73 (1.16–11.98)	

Adopted factors: age, sex, diabetes, systolic blood pressure, calcium, phosphorus, intact parathyroid hormone, glucose, and osteoprotegerin levels

*HD* hemodialysis, *OPG* osteoprotegerin, *PTH* parathyroid hormone

^¶^*p* for trend

*p < 0.05 is considered as statistically significant

**Fig. 1:**
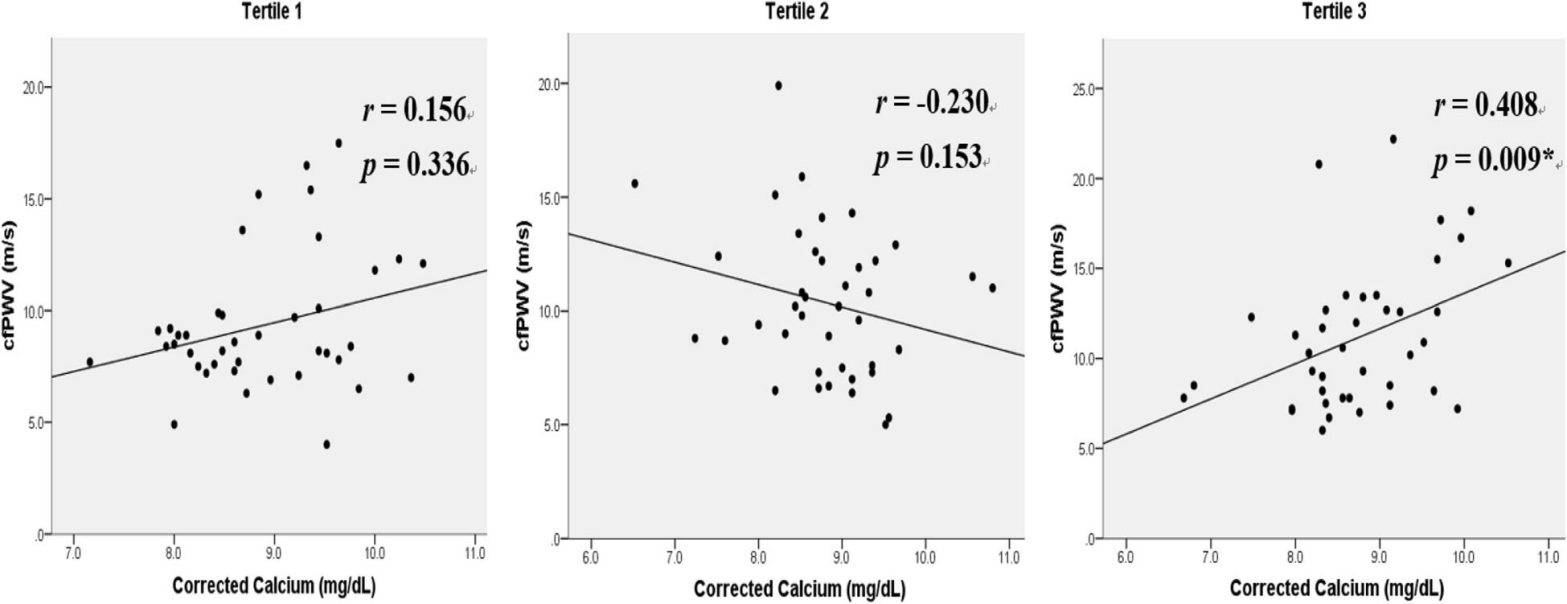
Correlation between serum calcium levels and carotid–femoral pulse wave velocity (cfPWV) values based on the tertiles of serum osteoprotegerin levels. Data of cfPWV showed skewed distribution, and their correlation with corrected serum calcium levels was analyzed using Spearman’s correlation analysis. *p <  0.05 is considered as statistically significant

Furthermore, we analyzed the correlation between corrected calcium levels and cfPWV values based on the tertiles of serum OPG levels, as shown in Fig. 1. The corrected calcium levels showed a positive correlation with cfPWV values in the tertile 3 group (*r* = 0.408, *p* = 0.009), but not in the tertile 1 or 2 group.

## Discussion

This study demonstrated that cfPWV was independently associated with DM and serum OPG levels in our sample of HD patients. In addition, older age, high serum OPG levels, DM, low iPTH levels, and high serum calcium levels were identified as independent predictors for high arterial stiffness. Moreover, in patients in the highest OPG tertile groups, serum calcium levels positively correlated with cfPWV values.

OPG is not only a well-known factor that is involved in the bone remodeling process, but it also plays an important role in vascular calcification. OPG is highly expressed in vascular smooth muscle cells and endothelial cells of arteries and has been regarded as a vascular protective factor by inhibiting vascular calcification; OPG-deficient mice were shown to exhibit an accelerated arterial calcification [15]. However, several observational studies have demonstrated that serum OPG levels appear to be elevated in patients with vascular damage and were considered as a strong vascular risk factor in a variety of different populations, including healthy people and patients with DM, hypertension, coronary artery disease, and CKD [12, 16–21]. In addition, elevated serum OPG levels were found to be associated with CV events and mortality in patients with ESRD [22, 23]. This paradoxical phenomenon may be partially explained by the compensatory response of serum OPG levels in mitigating further vascular calcification [5]. Consistent with previous studies, our patients in the high arterial stiffness group showed significantly higher serum OPG levels that, after being categorized into tertiles, demonstrated a significant trend with central arterial stiffness after complete adjustment of potential confounding factors, analyzed as either continuous or dichotomous outcome variable.

Hypercalcemia has been regarded as a promoter in the development of vascular calcification in patients with ESRD [4, 24]. In our study, we found that serum calcium levels were an independent predictor for arterial stiffness among our chronic HD patients. In addition, a positive correlation was found between serum calcium levels and cfPWV only in patients in the highest OPG tertile groups. These unique findings indicate that patients with high serum OPG levels may experience a greater influence of calcium load on central arterial stiffening.

Some studies have demonstrated that vascular calcification is accelerated in both high and low bone turnover disease [24–26]. In our study, patients with low serum iPTH levels, which indicated low bone remodeling, exhibited an increased risk of developing high arterial stiffness compared to those with iPTH levels in the normal range. However, the elevated serum iPTH levels were not significantly associated with high arterial stiffness in our study, which could be attributed to the limited sample size in this subgroup.

DM is associated with an increased activity of renin-angiotensin-aldosterone system, oxidative stress, formation of advanced glycation end-products (AGEs), enhanced expression of angiotensin type I receptor in vascular tissue, and impaired PI3-kinase–dependent signaling, all of which promote the development of wall hypertrophy and fibrosis [27, 28]. Therefore, patients with diabetic ESRD exhibit more advanced atherosclerotic changes of arteries that result in arterial stiffness [29]. In our study, up to two thirds of patients in the arterial stiffness group had DM. Moreover, DM was found to be the major predictor of arterial stiffness in our chronic HD patients. Such an association between DM and arterial stiffness in chronic HD patients has also been demonstrated in previous studies [29–32].

Several limitations must be acknowledged in our study. First, the sample size was relatively small. Second, measurements of blood pressure and cfPWV values before the HD session may be influenced by the hydration status. Third, we could not obtain the causal relationship between serum OPG levels and arterial stiffness in this cross-sectional study. Finally, we did not include certain medications in our analysis that may have an influence on arterial stiffness, such as phosphate binders and vitamin D analogs.

## Conclusions

Our study has demonstrated that, in addition to age, DM, hypercalcemia, and low serum iPTH levels, which are well-known factors contributing to arterial stiffness, a strong and positive correlation exists between serum OPG levels and arterial stiffness in patients with chronic HD. Moreover, positive correlations were found between serum calcium levels and cfPWV in patients in the highest OPG tertile groups, indicating that patients with high serum OPG levels may experience a significantly greater influence of calcium load on central arterial stiffening. Further studies are warranted to determine whether serum OPG is a surrogate marker or plays a causal role directly in mediating against vascular injury in patients with ESRD.

## Abbreviations

ACEIs: Angiotensin-converting enzyme inhibitors
AGEs: Advanced glycation end-products
ARBs: Angiotensin receptor blockers
BMI: Body mass index
BUN: Blood urea nitrogen
CCBs: Calcium channel blockers
cfPWV: Carotid–femoral pulse wave
CKD: Chronic kidney disease
CV: Cardiovascular
DM: Diabetes mellitus
ELISA: Enzyme-linked immunosorbent assay
ESRD: End-stage renal disease
HD: Hemodialysis
iPTH: Intact parathyroid hormone
OPG: Osteoprotegerin
RANKL: Receptor activator of nuclear factor kappa-B ligand
TRAIL: TNF-related apoptosis-inducing ligand

## References

[1] Gansevoort RT, Correa-Rotter R, Hemmelgarn BR, Jafar TH, Heerspink HJL, Mann JF, Matsushita K, Wen CP (2013). Chronic kidney disease and cardiovascular risk: epidemiology, mechanisms, and prevention. Lancet.

[2] de Jager DJ, Grootendorst DC, Jager KJ, van Dijk PC, Tomas LM, Ansell D, Collart F, Finne P, Heaf JG, De Meester J (2009). Cardiovascular and noncardiovascular mortality among patients starting dialysis. Jama.

[3] Foley RN, Parfrey PS, Sarnak MJ (1998). Epidemiology of cardiovascular disease in chronic renal disease. J Am Soc Nephrol.

[4] Chen NX, Moe SM (2015). Pathophysiology of vascular calcification. Current Osteoporosis Reports.

[5] Schlieper G, Schurgers L, Brandenburg V, Reutelingsperger C, Floege J (2016). Vascular calcification in chronic kidney disease: an update. Nephrol Dial Transplant.

[6] Moe SM, Chen NX (2008). Mechanisms of vascular calcification in chronic kidney disease. J Am Soc Nephrol.

[7] Rochette L, Meloux A, Rigal E, Zeller M, Cottin Y, Vergely C (2018). The role of osteoprotegerin in the crosstalk between vessels and bone: its potential utility as a marker of cardiometabolic diseases. Pharmacol Ther.

[8] Kazama JJ, Shigematsu T, Yano K, Tsuda E, Miura M, Iwasaki Y, Kawaguchi Y, Gejyo F, Kurokawa K, Fukagawa M (2002). Increased circulating levels of osteoclastogenesis inhibitory factor (osteoprotegerin) in patients with chronic renal failure. Am J Kidney Dis.

[9] Laurent S, Cockcroft J, Van Bortel L, Boutouyrie P, Giannattasio C, Hayoz D, Pannier B, Vlachopoulos C, Wilkinson I, Struijker-Boudier H (2006). Expert consensus document on arterial stiffness: methodological issues and clinical applications. Eur Heart J.

[10] Mancia G, Fagard R, Narkiewicz K, Redon J, Zanchetti A, Bohm M, Christiaens T, Cifkova R, De Backer G, Dominiczak A (2013). ESH/ESC guidelines for the management of arterial hypertension: the task force for the Management of Arterial Hypertension of the European Society of Hypertension (ESH) and of the European Society of Cardiology (ESC). Eur Heart J.

[11] London GM, Blacher J, Pannier B, Guerin AP, Marchais SJ, Safar ME (2001). Arterial wave reflections and survival in end-stage renal failure. Hypertension.

[12] Wang JH, Lee CJ, Chen ML, Yang CF, Chen YC, Hsu BG (2014). Association of serum osteoprotegerin levels with carotid-femoral pulse wave velocity in hypertensive patients. J Clin Hypertens (Greenwich).

[13] Massry SG, Coburn JW, Chertow GM, Hruska K, Langman C, Malluche H, Martin K, LM MC, JT MC, Moe S, Salusky IB (2003). K/DOQI clinical practice guidelines for bone metabolism and disease in chronic kidney disease. Am J Kidney Dis.

[14] Ketteler M, Block GA, Evenepoel P, Fukagawa M, Herzog CA, McCann L, Moe SM, Shroff R, Tonelli MA, Toussaint ND (2017). Executive summary of the 2017 KDIGO chronic kidney disease–mineral and bone disorder (CKD-MBD) guideline update: what’s changed and why it matters. Kidney Int.

[15] Bucay N, Sarosi I, Dunstan CR, Morony S, Tarpley J, Capparelli C, Scully S, Tan HL, Xu W, Lacey DL (1998). Osteoprotegerin-deficient mice develop early onset osteoporosis and arterial calcification. Genes Dev.

[16] Zagura M, Serg M, Kampus P, Zilmer M, Zilmer K, Eha J, Unt E, Lieberg J, Kals J (2010). Association of osteoprotegerin with aortic stiffness in patients with symptomatic peripheral artery disease and in healthy subjects. Am J Hypertens.

[17] Anand DV, Lahiri A, Lim E, Hopkins D, Corder R (2006). The relationship between plasma osteoprotegerin levels and coronary artery calcification in uncomplicated type 2 diabetic subjects. J Am Coll Cardiol.

[18] Scialla JJ, Leonard MB, Townsend RR, Appel L, Wolf M, Budoff MJ, Chen J, Lustigova E, Gadegbeku CA, Glenn M (2011). Correlates of osteoprotegerin and association with aortic pulse wave velocity in patients with chronic kidney disease. Clin J Am Soc Nephrol.

[19] Pateinakis P, Papagianni A, Douma S, Efstratiadis G, Memmos D (2013). Associations of fetuin-a and osteoprotegerin with arterial stiffness and early atherosclerosis in chronic hemodialysis patients. BMC Nephrol.

[20] Kim SM, Lee J, Ryu OH, Lee KW, Kim HY, Seo JA, Kim SG, Kim NH, Baik SH, Choi DS (2005). Serum osteoprotegerin levels are associated with inflammation and pulse wave velocity. Clin Endocrinol.

[21] Sigrist MK, McIntyre CW, Levin A, Er L (2009). Elevated osteoprotegerin is associated with all-cause mortality in CKD stage 4 and 5 patients in addition to vascular calcification. Nephrol Dial Transplant.

[22] Morena M, Terrier N, Jaussent I, Leray-Moragues H, Chalabi L, Rivory JP, Maurice F, Delcourt C, Cristol JP, Canaud B (2006). Plasma osteoprotegerin is associated with mortality in hemodialysis patients. J Am Soc Nephrol.

[23] Lee JE, Kim HJ, Moon SJ, Nam JS, Kim JK, Kim SK, Yun GY, Ha SK, Park HC (2013). Serum osteoprotegerin is associated with vascular stiffness and the onset of new cardiovascular events in hemodialysis patients. Korean J Intern Med.

[24] London GM, Marchais SJ, Guérin AP, Boutouyrie P, Métivier F, de Vernejoul M-C (2008). Association of Bone Activity, calcium load, aortic stiffness, and calcifications in ESRD. J Am Soc Nephrol.

[25] Persy V, D’Haese P (2009). Vascular calcification and bone disease: the calcification paradox. Trends Mol Med.

[26] London GM, Marty C, Marchais SJ, Guerin AP, Metivier F, de Vernejoul M-C (2004). Arterial calcifications and bone Histomorphometry in end-stage renal disease. J Am Soc Nephrol.

[27] Zieman SJ, Melenovsky V, Kass DA (2005). Mechanisms, pathophysiology, and therapy of arterial stiffness. Arterioscler Thromb Vasc Biol.

[28] Cooper ME, Bonnet F, Oldfield M, Jandeleit-Dahm K (2001). Mechanisms of diabetic vasculopathy: an overview. Am J Hypertens.

[29] Shoji T, Emoto M, Shinohara K, Kakiya R, Tsujimoto Y, Kishimoto H, Ishimura E, Tabata T, Nishizawa Y (2001). Aortic stiffness, and cardiovascular mortality in end-stage renal disease. J Am Soc Nephrol.

[30] Aoun S, Blacher J, Safar ME, Mourad JJ (2001). Diabetes mellitus and renal failure: effects on large artery stiffness. J Hum Hypertens.

[31] Cameron JD, Bulpitt CJ, Pinto ES, Rajkumar C (2003). The aging of elastic and muscular arteries: a comparison of diabetic and nondiabetic subjects. Diabetes care.

[32] Strain WD, Chaturvedi N, Dockery F, Shiff R, Shore AC, Bulpitt CJ, Rajkumar C (2006). Increased arterial stiffness in Europeans and African Caribbeans with type 2 diabetes cannot be accounted for by conventional cardiovascular risk factors. Am J Hypertens.

